# Factors for Prolonged Pain and Restriction of Movement Following Hemiepiphysiodesis Plating for the Correction of Lower Limb Malalignment in the Frontal Plane: An Explorative Analysis

**DOI:** 10.3390/children10040686

**Published:** 2023-04-04

**Authors:** Sebastian Braun, Marco Brenneis, Andrea Meurer, Jana Holder, Felix Stief

**Affiliations:** 1 Department of Orthopedics (Friedrichsheim), University Hospital Frankfurt, Goethe University, 60528 Frankfurt am Main, Germany; 2 Medical Park St. Hubertus Klinik, 83707 Bad Wiessee, Germany; 3 Department of Sport and Exercise Science, University of Salzburg, 5020 Salzburg, Austria; 4 Dr. Rolf M. Schwiete Research Unit for Osteoarthritis, Department of Orthopedics (Friedrichsheim), University Hospital Frankfurt, Goethe University, 60528 Frankfurt am Main, Germany

**Keywords:** lower limb deformities, leg axis malalignment, postoperative pain, postoperative complications, pediatric orthopedic, implant-mediated growth guidance, tension band plate, hemiepiphysiodesis plate

## Abstract

The correction of valgus leg malalignment in children using implant-mediated growth guidance is widely used and effective. Despite the minimal invasive character of the procedure, a relevant number of patients sustain prolonged pain and limited mobility after temporary hemiepiphysiodesis. Our aim was to investigate implant-associated risk factors (such as implant position and screw angulation), surgical- or anesthesia-related risk factors (such as type of anesthesia, use, and duration), and pressure of tourniquet or duration of surgery for these complications. Thirty-four skeletally immature patients with idiopathic valgus deformities undergoing hemiepiphysiodesis plating from October 2018–July 2022 were enrolled in this retrospective study. Participants were divided into groups with and without prolonged complications (persistent pain, limited mobility of the operated knee between five weeks and six months) after surgery. Twenty-two patients (65%) had no notable complications, while twelve patients (35%) had prolonged complications. Both groups differed significantly in plate position relative to physis (*p* = 0.049). In addition, both groups showed significant differences in the distribution of implant location (*p* = 0.016). Group 1 had a shorter duration of surgery than group 2 (32 min vs. 38 min, *p* = 0.032) and a lower tourniquet pressure (250 mmHg vs. 270 mmHg, *p* = 0.019). In conclusion, simultaneous plate implantation at the femur and tibia and metaphyseal plate positioning resulted in prolonged pain and a delay of function. In addition, the amplitude of tourniquet pressure or duration of surgery could play a factor.

## 1. Introduction

The correction of axial lower limb malalignment in the frontal plane in children and adolescents by implant-mediated growth guidance with hemiepiphysiodesis plates for temporary hemiepiphysiodesis is a common and effective pediatric orthopedic procedure [1,2]. In contrast to the higher initial compression force of staples, hemiepiphysiodesis plates have a lower risk of physis fusion compared to these rigid staples [3,4,5]. The risk of extrusion or dislocation is lower in hemiepiphysiodesis plates because of their screws, and with their longer moment arm, faster correction rate is postulated [6]. The surgical procedure itself is minimally invasive compared to corrective osteotomies for angular deformities and has very low approach morbidity [7]. Nevertheless, the procedure appears to be associated with prolonged postoperative pain and reduced mobility and activity [8,9].

The current literature on hemiepiphysiodesis plating focuses on clinical outcomes, particularly its effectiveness as a guided growth system for correcting deformities, the speed of correction, and the incidence of rebound compared with other procedures [3,10,11]. However, Gregoire et al. [8] showed that 38% of patients still needed to take pain medication four weeks after temporary hemiepiphysiodesis and 65% did not return to previous activities during that time. Another study also showed a delay in postoperative return to full function [9]. There have been few reports to date with regard to surgical and implant-related risk factors for prolonged recovery with increased pain and limited postoperative knee range of motion. To the best of our knowledge, no study has investigated the cause of such complications. Therefore, the aim of this explorative study was to investigate implant-associated, surgery- or anesthesia-related, and other risk factors for postoperative complications. Our main hypothesis was that implant position is a factor in functional delay and prolonged pain after hemiepiphysiodesis plating. In particular, we hypothesized that the implantation angle of the screws has an influence on the incidence and duration of postoperative pain. We assumed that a divergent angle would result in more initial pain than parallel screws due to increased pressure and compression force on the physis.

## 2. Materials and Methods

### 2.1. Patients

Children and adolescents with idiopathic valgus deformities without other comorbidities were prospectively enrolled at our institution between October 2018 and July 2022. The indication for implant-mediated growth guidance with hemiepiphysiodesis plating was set for skeletally immature patients with a pathological idiopathic valgus alignment deformity (mechanical axis deviation (MAD) of >10 mm and/or mechanical femorotibial angle (MFA) of >3°) of one or both lower extremities [12]. To decide whether the angular deformity originated in the femur or tibia, the mechanical lateral distal femur angle (mLDFA) and mechanical medial proximal tibia angle (mMPTA) were determined, and the indication for surgery on the femur, tibia, or both segments was made according to the pathological joint surface angles (physiological values for mLDFA 88° +/− 2.5° and for mMPTA 87° +/− 2.5°) [13]. Eight-Plates (Orthofix, Lewisville, TX, USA) or Pedi-Plates (Orthopediatrics Inc., Warsaw, IN, USA) were used in this study. The same surgical technique was performed in all patients. The plates were inserted through a minimally invasive technique in an open procedure under fluoroscopic control. Local anesthesia was not applied to any patient.

Immediately after surgery, patients were allowed to resume full weight bearing, but sports and high impact activities were not permitted until four weeks postoperatively. Patients were discharged from the hospital after achieving 90° knee flexion and returned for follow-up visits at four weeks, three months, and then at three-month intervals until the leg axis was corrected (successful growth guidance was determined by an MFA of 0° +/− 2° or an MAD of 0 mm +/− 6 mm) and the plates were removed. Physical therapy was performed daily during the inpatient hospital stay (usually 1–3 days). Braces were not applied. 

**Figure 1:**
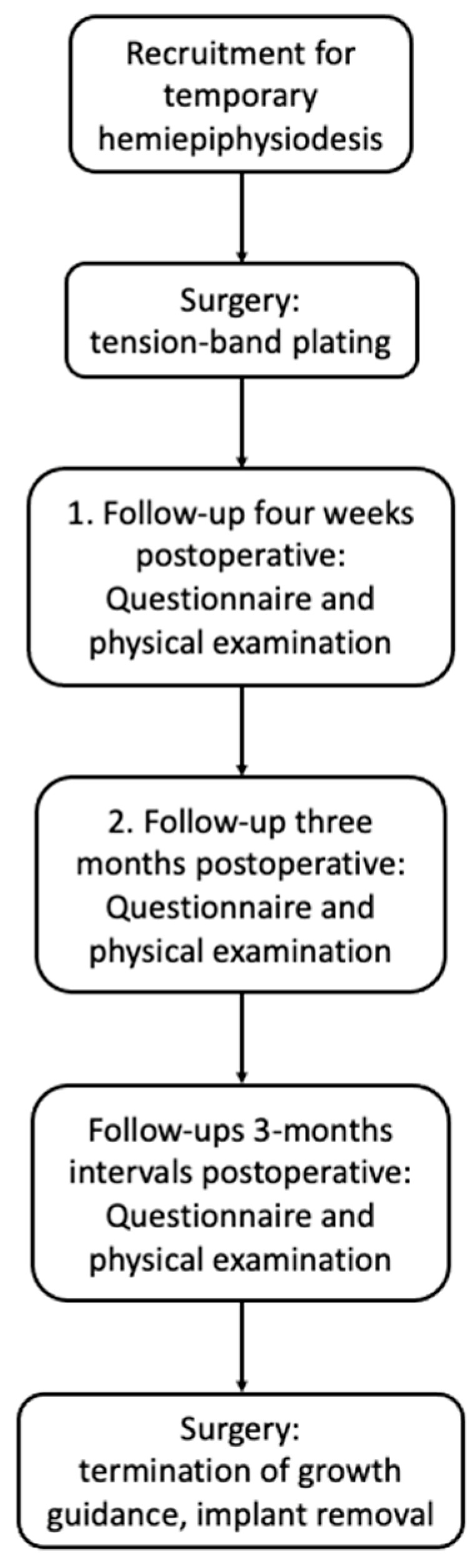
Flow chart of the study design.

Patients were included in this study only if they completed a postoperative questionnaire asking about the presence of pain (yes or no) and limitation of motion in the operated knees during routine postoperative appointments at one, three, and six months after implantation of the plates. In addition to the questionnaire, a clinical examination was performed in each case to determine if there was any limitation of the knee joint movement and range of motion (see Figure 1). 

Exclusion criteria were: rheumatoid arthritis, anterior cruciate ligament deficiency, neuromuscular disorders, achondroplasia or hypochondroplasia, sagittal plane deformities (genu pro- and recurvatum), flexion contractures in the hip or knee joint, leg length discrepancy of >10 mm, avascular necrosis of the femoral head or knee condyles, history of severe trauma or sport injury to the lower extremities, knee surgery within 12 months before enrollment in this study, chronic joint infections, or prior intraarticular corticosteroid injections.

Patients were divided into two groups: one with no particular complications after surgery (no complications group) and the other with marked complications persisting over a period of at least five weeks after surgery. Marked complications were defined as persistent pain and limited mobility of the operated knee after hemiepiphysiodesis plating between five weeks and six months. Participants and their parents provided written informed consent to participate in this study, which was approved by the local ethics committee (182/16) and in accordance with the Helsinki Declaration (date of approval: 30 December 2015). This study was registered with DRKS (German Clinical Trials Register) under the number DRKS00010296.

### 2.2. Radiographic Measurements

**Figure 2:**
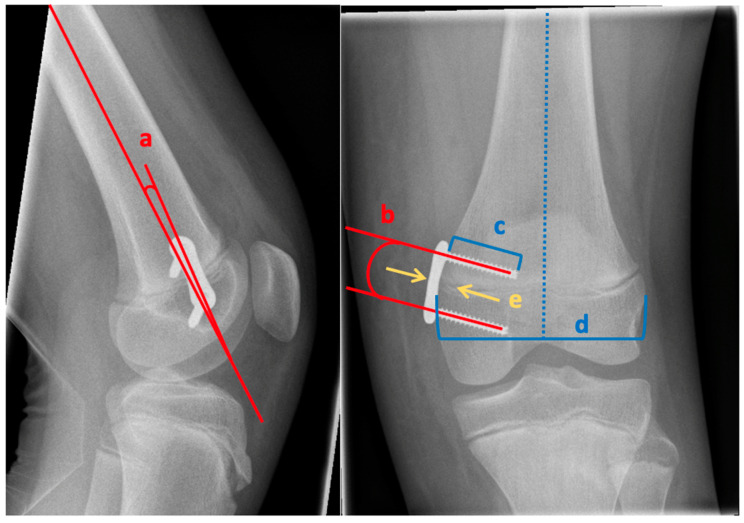
Radiological assessment: a—angle between the implant and femur axis, b—angle between the two screws, c—length of the screw, d—width of the epiphysis (greatest width of the epiphysis perpendicular to the axis of the femur), e—relation of the center of the plate to the physis (left arrow pointing at the center of the plate and right arrow pointing at the center of the physis).

**Figure 3:**
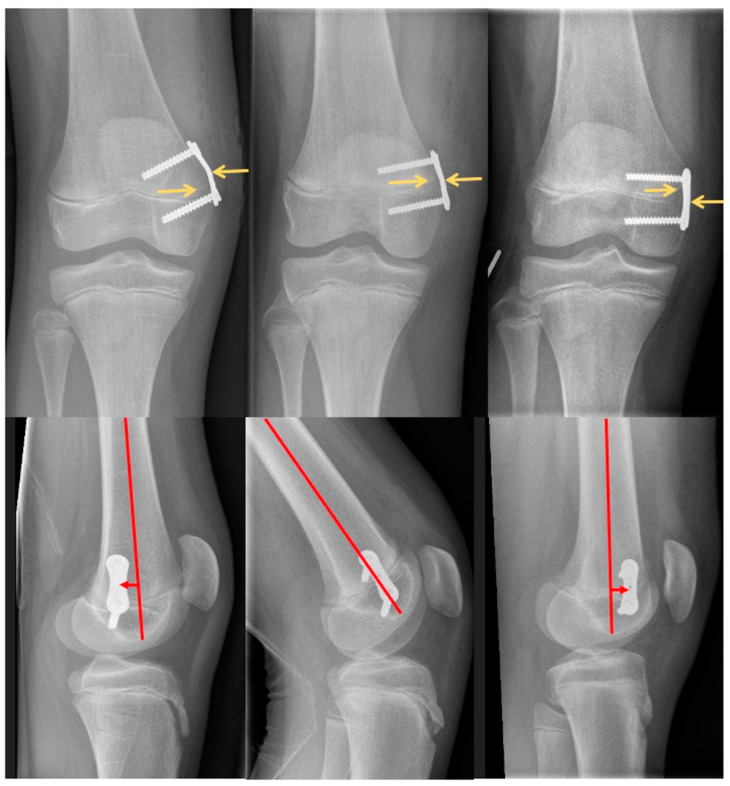
Radiological evaluation of the plate position: **top** row—plate position in relation to the physis, left arrow pointing at the center of the physis and right arrow pointing at the center of the plate) (**left** image: metaphyseal position, **center** image: centered position, **right** image: epiphyseal position); **bottom** row—plate position in relation to the center of the shaft axis, arrow pointing in the direction of insertion (**left** image: posterior position, **center** image: centered position, **right** image: anterior position).

Implant position was analyzed on postoperative lateral and anterior–posterior X-rays of the knee. Data were obtained on the insertion site (femur and/or tibia), implant size, angulation of the plate in relation to the shaft axis, angulation of the screws at time of implantation (parallel, divergent, convergent), ratio between screw length and epiphyseal width (Figure 2), plate position in relation to the center of the shaft axis, and plate position in relation to the physis (Figure 3). A 25.4 mm diameter metal ball was placed adjacent to the knee and served as a reference for determining the individual magnification factor. Radiographic measurements were performed by the same orthopedic surgeon (SB) with a commercially available templating program, mediCAD^®^ (version 5.98; Hectec, Niederviehbach, Germany).

Each patient’s medical record was reviewed for information on age, sex, diagnosis with relevant leg malalignment and joint angle parameters (MAD, MFA, joint line convergence angle), as well as the date of implantation and surgery to remove the hemiepiphysiodesis plates. In addition, data were extracted from the surgical report: type of anesthesia, use, duration, and pressure amplitude of tourniquet, and duration of surgery. All patients received general anesthesia combined with regional anesthesia (peripheral neuraxial blocks: single-shot adductor canal block, femoralis block, or psoas compartment block), followed by a standardized analgetic postoperative treatment with a dose of ibuprofen or acetaminophen adjusted to the patients’ body weight.

### 2.3. Statistical Analysis

The Shapiro–Wilk test was used to test the normal distribution of the parameters analyzed. Non-parametric independent variables were compared with the Mann–Whitney U test. For normally distributed data, unpaired two-sided Student’s *t*-tests were used to assess statistical significance between two sample means. Differences between nominally distributed variables were analyzed using the chi-square test or Fisher’s exact test when the expected count was less than 5. For this explorative analysis, no confirmatory primary endpoint/hierarchical test approach was selected. The significance level for all statistical tests was set at *p* ≤ 0.05. No α-adjustments for multiple testing were applied. Statistical data analysis was performed using SPSS (version 29, IBM Corporation, New York, NY, USA). 

## 3. Results

Thirty-four patients met the criteria for evaluation. Group 1 (no complications) consisted of 22 patients, 55% of whom were female and had a mean age at surgery of 12.5 years (range 11.1–15.7). Group 2 (complications) included 12 patients (25% females) with a mean age at surgery of 13.2 years (range 11.5–14.3). Accordingly, 35% of patients had persistent pain and limited mobility of the operated knee for more than four weeks after hemiepiphysiodesis plating. Table 1 shows the anthropometric data and radiological extent of angular deformity before surgery. Patients had idiopathic genu valgum deformity with a mean MFA of 5.7° (1.8) and MAD of −19.4 (5.7) mm in group 1 and 5.3° (2.1) and −19.2 (7.9) mm in group 2. There were no statistical differences in anthropometric data, extent of the initial deformity, and duration of guided growth between the two groups. All patients experienced no complications other than the aforementioned mobility limitations and prolonged pain in the operated knee joint.

Both groups differed significantly in plate positioning relative to the physis (*p* = 0.049). In group 1, 77.7% of plates were placed centered, 16.7% closer to the epiphysis, and 5.6% closer to the metaphysis. In group 2, 77.8% of plates were placed centered, 22.2% closer to metaphysis, and no plate was placed closer to epiphysis (Table 2). There was no difference in plate positioning in relation to the shaft axis (parallel, in flexion, in extension) (*p* = 0.312), plate position in relation to the center of the shaft axis (*p* = 0.388), angulation of the screws at the time of implantation (parallel, convergent, or divergent) (*p* = 0.264), and screw length/epiphysis width ratio (*p* = 0.797).

In group 1, 18 patients (82%) underwent bilateral surgery, compared to 10 patients (83%) in group 2. Four patients (18%) in group 1 and two patients (17%) in group 2 underwent unilateral surgery. There were significant differences in the distribution of implant localization between these two groups (*p* = 0.016). In 90.2% of patients in group 1, plates were inserted at the distal femur, in 4.9% of patients at the proximal tibia, and 4.9% of patients at the distal femur and proximal tibia. In group 2, plates were inserted at the distal femur in 69.2% of patients, and at the distal femur and proximal tibia in 30.8% of patients. In no patients were they placed only at the proximal tibia (Table 3).

## 4. Discussion

With this study, we aimed to investigate implant-associated, surgery- or anesthesia-related, and other risk factors for complications regarding prolonged pain and limited range of motion postoperatively in children and adolescents with an idiopathic valgus deformity treated with temporary hemiepiphysiodesis. We hypothesized that implant insertion and position are related to function and pain after hemiepiphysiodesis plating and that screw implantation angle is associated with postoperative complications.

The results of the present study indicate that neither the initial extent of lower limb malalignment nor the timing of implant-mediated growth guidance (time from implant placement to implant removal) appear to be associated with a high number of prolonged postoperative complications. Anthropometric characteristics such as body mass index and age also had no statistical effect on the rate of prolonged pain after surgery. Fillingham et al. [9] showed that patients older than 11 years of age at the time of implantation tended to have a greater delay in function. The results of the present study could not exactly confirm this finding because all our patients were older than 11 years and there was no difference in the age distribution of the two groups. However, because 35% of our patients had prolonged pain and functional delay, a comparison with a younger cohort of patients would be useful to support their findings.

**Figure 4:**
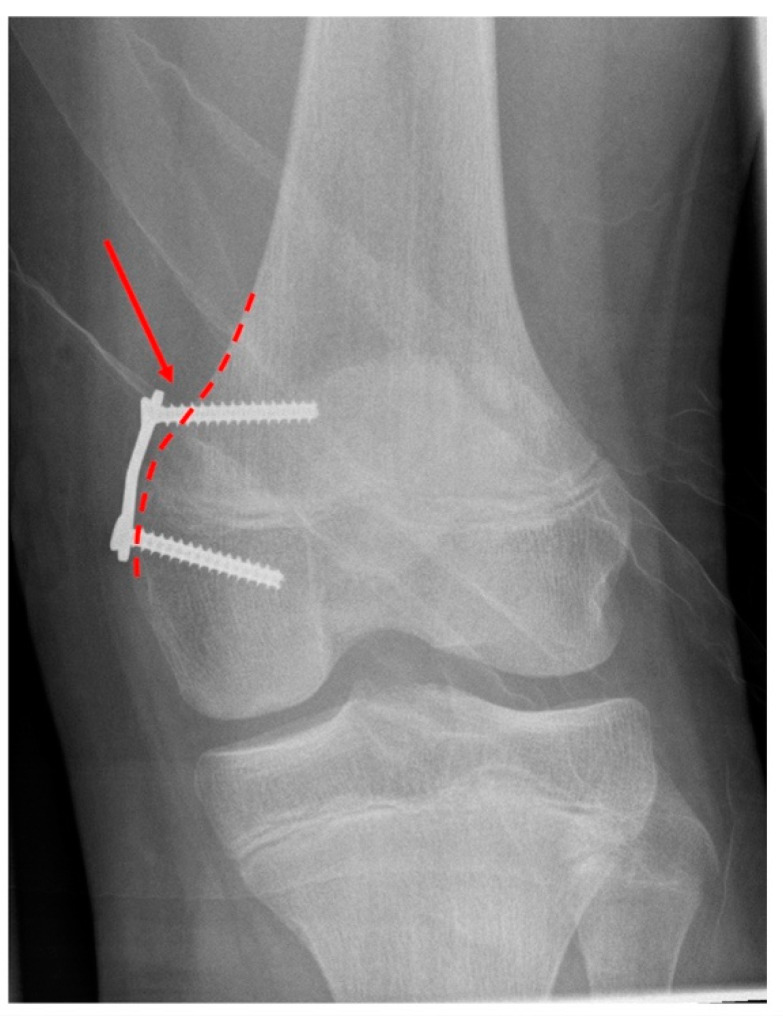
Metaphyseal plate position with pull-out and loss of initial contouring of the metaphyseal screw. Dashed line outlines the bone structure and cortical contour. Arrow pointing at the pull-out and loss of initial contouring resulting in lift-off of the plate at the metaphyseal end.

In the present study, we demonstrated that the precise positioning of the plates in relation to the physis may have an impact on the postoperative complication rate. In this context, a metaphyseal position of the plate could lead to increased and prolonged postoperative pain and functional limitations. We suspect that periosteal preparation further proximally (femoral) or distally (tibial) leads to increased muscle dissection, which could result in a prolonged healing process with pain and limited range of motion. In addition, 25% of patients from group 2 (complications) had a protruding plate (Figure 4). A metaphyseal positioned plate does not necessarily have to protrude, as care should be taken to ensure contour-fit insertion onto the corticalis of the femur during implantation. Nevertheless, a metaphyseally placed plate may protrude more easily. In this case, soft tissue could interpose between the bone and the plate and cause pain symptoms. Therefore, surgeons must be careful to position the implant correctly to avoid such complications.

We hypothesized that the implantation angle of the screws has an influence on postoperative pain due to the compression force exerted on the physis [14]. Here, the existing literature is divided as to whether a divergent angle of the screws increases the correction rate and exerts more pressure on the epiphyseal joint. Burghardt et al. [15] suggested that the rate of correction is slower when the screws are initially inserted in parallel, and the correction accelerates when the screws become more divergent as growth progresses. In contrast, Schoenleber et al. [16] demonstrated in a biomechanical study that initial parallel screw positioning results in faster correction compared to divergent screws. Eltayeby et al. [17] showed that the initial screw angle ranging from parallel to 30° in divergence had no significant effect on the speed of correction during hemiepiphysiodesis. Assuming that screw angle does indeed result in an altered correction velocity due to pressure and compression force changes, these altered pressures could also result in greater pain and limitation of knee range of motion. To our knowledge, no previous study has investigated the effect of screw angle on postoperative knee pain and range of motion after hemiepiphysiodesis plating. In our study, we were able to demonstrate that the initial screw implantation angle did not make a difference between the two groups and, therefore, was unlikely to be responsible for persistent pain symptoms or limitation of knee range of motion. Consequently, our hypothesis regarding the angulation of the screws has to be rejected.

Fillingham et al. [9] showed that plating bilaterally versus unilaterally, femur versus tibia, or the number of implants used for implant-mediated growth guidance conferred a greater risk of functional delay. Given the predominance of plates in the distal femur (only 2 of 66 implants were placed at the proximal tibia) in our patients, our study is not sufficiently powered to detect a difference based on location. Nevertheless, in accordance with the study by Fillingham et al. [9], the results of the present study showed that patients with simultaneous tibial and femoral implantation had higher complication rates (*p* = 0.016).

Regional anesthesia with peripheral neuraxial blocks is commonly performed in pediatric orthopedic surgery [18]. They are associated with a very low risk of complications [19,20] and have the same effect regardless of the different types of regional anesthesia [21]. In the present study, we demonstrated that the different types of regional anesthesia (adductor canal block, femoralis block, psoas compartment block) combined with general anesthesia showed no difference in postoperative pain or a delay of function after surgery. However, in the present study, patients tended to have a higher complication rate and pain after implantation of hemiepiphysiodesis plates at higher tourniquet pressures. Accordingly, Hanna et al. [22] found a reduction of opioid consumption in the postoperative period by avoiding the use of tourniquet in pediatric patients with lower limb surgery. Another study from Kamath et al. [23] found that the incidence of tourniquet pain was directly proportional to the duration of tourniquet use but not to the amplitude of tourniquet inflation pressure. In their study, 7.7% of patients experienced tourniquet pain after a surgery that lasted less than 60 min, compared with 35.8% after a procedure that lasted longer than 60 min. Tourniquet pain is described as a poorly localized, dull, tight, aching sensation at the site where the tourniquet is applied [24]. In the present study, the duration of the procedure did not exceed 60 min in either group (group 1: mean 32 min: group 2: mean 38 min). Therefore, it is difficult to distinguish between surgery-related pain and tourniquet-related pain in our patient group. Lieberman et al. [25] investigated tourniquet pressures with values of 50 mmHg above the occlusion pressure measured by Doppler. According to their work, lower tourniquet pressures (176.7 +/− 28.7 mmHg, range 140 to 250 mmHg) can maintain adequate hemostasis in a lower extremity surgery in pediatric patients [25]. Consistent with our data, we can recommend tourniquet pressures of no more than 250 mmHg to preclude persistent complications and impairments after surgery.

In children and adolescents, symptoms such as persistent pain, restricted mobility, and range of motion after surgery may also be caused by the complex regional pain syndrome (CRPS). In affected children, the peak of CRPS type I, the cause of which has not yet been conclusively identified but which can occur after surgery, appears to be at the age of 13 years. Chronic pain, generally unilateral and limited to the extremities, autonomic and motor dysfunction, and trophic disturbances are the main symptoms of CRPS type 1 [26]. The diagnosis of CRPS type 1 is typically made clinically and is based on the Budapest diagnostic criteria [27]. However, the diagnosis remains difficult given the lack of validated diagnostic tests and the difficulties in differential diagnosis [26]. The children in this study were not explicitly screened for this diagnosis.

From an anatomical point of view, the cause of prolonged postoperative pain can be assumed to be the injury of small cutaneous nerves due to the open procedure itself. The medial and lateral part of the knee are innervated by different nerves. The saphenous nerve is the primary cutaneous nerve that supplies sensation to the skin over the medial knee. The superior medial genicular nerve also supplies sensation to the skin over the medial knee, both are branches of the femoral nerve. The superior lateral genicular nerve is a branch of the common peroneal nerve (a branch of the sciatic nerve) which supplies sensation to the lateral aspect of the knee joint capsule and the skin over the lateral knee [28]. Even during such a minimally invasive procedure as the implantation of temporary hemiepiphysiodesis, these small nerve branches can be injured. One solution can be the percutaneous insertion of the plates and screws through two 6 mm incisions, which, according to a large amount of experience in percutaneous insertion, reduces this frequently observed, undesirable complication after surgery [29].

When interpreting the results of the present study, its limitations should be considered. The influence of the surgeon on the success of surgery cannot be demonstrated with certainty because of the large number of surgeons involved. In addition, it should be noted as a limitation that the postoperative assessment and questionnaires did not explicitly differentiate between pain at the knee or at the insertion site of the plate and the application of the tourniquet to the proximal thigh. Lastly, no adjustment for multiple testing was applied, thus overall, our results and their interpretation have an exploratory character and should be treated with some caution as they can be due to coincidence.

## 5. Conclusions

In conclusion, 35% of patients had more postoperative limitations than expected, with persistent pain and limited mobility of the operated knee after hemiepiphysiodesis plating between five weeks and six months. In implant-mediated growth guidance with hemiepiphysiodesis plating for temporary hemiepiphysiodesis, the simultaneous implantation of the plates at the femur and tibia and the metaphyseal positioning of the plates may result in prolonged pain symptoms and a delay of function. In addition, surgery-related factors such as the amplitude of tourniquet inflation pressure or the duration of surgery, could play a role in the development of a poor outcome. In contrast, neither the initial extent of lower limb malalignment nor the timing of implant-mediated growth guidance (time from implantation to implant removal) appear to be associated with longer-lasting postoperative complications. Body mass index and age also had no effect on the complication rate after surgery.

## Figures and Tables

**Table 1:** Group differences.

Patients (n = 34)			
Parameter	Group 1 No Complications	Group 2 Complications	*p*-Value
Patient characteristics			
Number of subjects	22 (65%)	12 (35%)	
Sex (female/male)	12 (55%)/10 (45%)	3 (25%)/9 (75%)	0.09
Age at surgery (years, months)	12.5 (1.1)	13.2 (1.0)	0.11
Height (m)	1.61 (0.10)	1.68 (0.13)	0.06
Body mass index (kg/m^2^)	22.1 (3.8)	22.5 (3.6)	0.78
Extent of deformity (X-ray)			
Mechanical axis deviation (MAD) (mm)	−19.4 (5.7)	−19.2 (7.9)	0.90
Mechanical femorotibial angle (MFA) (°)	−5.7 (1.8)	−5.3 (2.1)	0.45
Joint-line conversion angle (°) *	1.0 (1.0–2.0)	1.0 (1.0–2.0)	0.32
Duration of guided growth (weeks) *	40.1 (34.3–51.7)	41.4 (41.0–46.9)	0.61

Parametric data: mean with standard deviation in parenthesis. * Non-parametric data: median with interquartile range in parenthesis. Mechanical axis deviation: negative values describe a valgus alignment. Mechanical femorotibial angle: negative values describe a valgus alignment.

**Table 2:** Radiographic evaluation of implant positioning/characteristics.

Parameter	Group 1 No Complications	Group 2 Complications	*p*-Value
Plate position in relation to the physis			0.049
centered	28 (77.7%)	14 (77.8%)	
epiphyseal	6 (16.7%)	0 (0.0%)	
metaphyseal	2 (5.6%)	4 (22.2%)	
Plate position in relation to the shaft axis of femur/tibia (°)	2.1 (11.2)	5.9 (13.5)	0.31
Plate position in relation to the center of the shaft axis of femur/tibia			0.39
centered	18 (50.0%)	10 (71.4%)	
posterior	12 (33.3%)	2 (14.3%)	
anterior	6 (16.7%)	2 (14.3%)	
Angulation of the two screws (°)	0.9 (5.3)	2.8 (6.3)	0.26
Epiphyseal width/diameter (cm)	8.2 (1.0)	8.4 (0.9)	0.44
Length of the screw (cm)	2.7 (0.4)	2.8 (0.4)	0.60
Ratio screw length/epiphyseal width	0.34 (0.03)	0.33 (0.03)	0.80

Parametric data: mean with standard deviation in parenthesis. Positive values of plate position in relation to the shaft axis of femur/tibia mean plate insertion in flexion, negative values mean plate insertion in extension. Positive values of angulation of the screws mean divergent screws, negative values mean convergent screws.

**Table 3:** Types of implant and implant localization.

Parameter	Group 1 Complications	Group 2 Complications	*p*-Value
Procedures/Types of implant			
Unilateral surgery (number of patients)	4 (18%)	2 (17%)	
Bilateral surgery (number of patients)	18 (82%)	10 (83%)	
Number of implants	41	26	
Pedi-Plate (number)	37 (90%)	26 (100%)	
Eight-Plate™ (number)	4 (10%)	0 (0%)	
Implant localization			0.016
Medial distal femur	37 (90.2%)	18 (69.2%)	
Medial proximal tibia	2 (4.9%)	0 (0.0%)	
Medial distal femur and medial proximal tibia	2 (4.9%)	8 (30.8%)	

Parametric data: mean with standard deviation in parenthesis. There was no difference in the type of additive regional anesthesia (*p* = 0.060). All patients had general anesthesia and most also had additional regional anesthesia (see Table 4). Group 1 had a shorter duration of surgery (32 min vs. 38 min, *p* = 0.032) and a lower pressure of tourniquet (250 mmHg vs. 270 mmHg, *p* = 0.019) compared with group 2. The duration of tourniquet inflation showed no significant difference (*p* = 0.162).

**Table 4:** Group differences.

Parameter	Group 1 Complications	Group 2 Complications	*p*-Value
Type of anesthesia			0.06
Adductor canal block + general anesthesia	27 (69.3%)	17 (77.3%)	
Femoralis block + general anesthesia	8 (20.5%)	0 (0.0%)	
Psoas compartment block + general anesthesia	2 (5.1%)	3 (13.6%)	
General anesthesia	2 (5.1%)	2 (9.1%)	
Surgery characteristics			
Tourniquet pressure (mmHg) *	250 (250–265)	270 (250–280)	0.019
Tourniquet inflation (minutes)	34 (9)	37 (15)	0.16
Duration surgery (minutes)	32 (10)	38 (13)	0.032

Parametric data: mean with standard deviation in parenthesis. * Non-parametric data: median with interquartile range in parenthesis.

## Data Availability

The data presented in this study are available upon request from the corresponding author.
